# Description on two species of genus *Platythomisus* (Araneae, Thomisidae) from China and Singapore

**DOI:** 10.3897/zookeys.852.34436

**Published:** 2019-06-05

**Authors:** Yejie Lin, Joseph K.H. Koh, Lili Shao, Shuqiang Li

**Affiliations:** 1 College of Life Science, Langfang Normal University, Langfang 065000, Hebei Province, China Langfang Normal University Langfang China; 2 National Biodiversity Centre, National Parks Board, 259598, Singapore National Biodiversity Centre Singapore Singapore; 3 Institute of Zoology, Chinese Academy of Sciences, Beijing 100101, China Institute of Zoology, Chinese Academy of Sciences Beijing China

**Keywords:** African region, new species, Oriental region, taxonomy, type species

## Abstract

Two species of the genus *Platythomisus* Doleschall, 1859 are studied: *P.xiandao* Lin & Li, **sp. nov.** is described based on male and female specimens from Yunnan, China, and *P.octomaculatus* (C. L. Koch, 1845), the type species of the genus, is redescribed based on female specimens from Singapore. Its male, also from Singapore, is described for the first time.

## Introduction

The spider genus *Platythomisus* Doleschall, 1859 includes 13 species, of which nine are known in the African region and four are distributed in the Oriental region, viz., *P.jucundus* Thorell, 1894 (♂, Indonesia), *P.octomaculatus* (C. L. Koch, 1845) (♀, Brunei, India, Indonesia, Malaysia, Singapore, Thailand), *P.quadrimaculatus* Hasselt, 1882 (juvenile, Indonesia), and *P.sudeepi* Biswas, 1977 (♂♀, India, Sri Lanka) (WSC 2019; Li and Quan 2017).

We have since found matched pairs of two *Platythomisus* species in collections from Singapore and China. The discovery of one of these species, *Platythomisusxiandao* Lin & Li sp. nov., from Yunnan represents the first record of this genus in China. Furthermore, specimens of *P.octomaculatus* (C. L. Koch, 1845) from Singapore have allowed us to provide the first description of the male of the species.

## Material and methods

All specimens were preserved in 80% ethanol. Dissected genitalia were cleared in warm 10% potassium hydroxide (KOH) solution before study. Specimens were examined under a LEICA M205C stereomicroscope. Photomicroscopy images were taken with an Olympus C7070 zoom digital camera (7.1 megapixels). Laboratory habitus photographs were taken with a Canon 5D Mark III digital camera equipped with a Canon MP-E 65 mm lens. Photos were stacked with Helicon Focus (version 6.7.1) or Zerene Stacker (version 1.04) and processed in Adobe Photoshop CC2018. Field photographs were taken with a Nikon D800E with a Tamron 90 mm macro lens.

All measurements are in millimeters and were obtained with a LEICA M205C stereomicroscope. Eye sizes are measured as the maximum diameter from either the dorsal or frontal view. Leg measurements are given as follows: total length (femur, patella, tibia, metatarsus, tarsus). The terminology used in the text and figures follows Ono (1988). Distribution maps were generated using ArcMap software (version 10.2).

The types of *Platythomisusxiandao* Lin & Li, sp. nov. are deposited at the Institute of Zoology, Chinese Academy of Sciences in Beijing (**IZCAS**). The voucher specimens of *P.octomaculatus* of this study are kept at the Lee Kong Chian Natural History Museum, National University of Singapore (**LKCNHM**).

To confirm the species delimitation, a fragment of the cytochrome c oxidase subunit I (COI) was amplified and sequenced. Primer sets for the PCR and cycle sequencing reactions in this study are from Folmer et al. (1994). The GenBank accession numbers are provided in Table 1. MEGA7.0.16 (Kumar et al. 2016) was used for subsequent manual adjustment of the sequences and calculation of pairwise comparisons of uncorrected K2P-distances. COI sequences of *Thomisus* Walckenaer, 1805 were also obtained from GenBank to calculate intraspecific genetic distance.

**Table 1.** The accession numbers for two species in this paper.

Species	Length (bp)	GenBank accession number
* Platythomisus octomaculatus *	647 bp	MK774520
*Platythomisusxiandao* sp. nov.	647 bp	MK774521

Abbreviations: **ALE** anterior lateral eyes, **AME** anterior median eyes, **PLE** posterior lateral eyes, **PME** posterior median eyes, **E** embolus, **ITA** intermediate tibial apophysis, **RTA** retrolateral tibial apophysis, **VTA** ventral tibial apophysis, **At** atrium, **CD** copulatory duct, **S** spermathecae.

## Taxonomy

### Family Thomisidae Sundevall, 1833

#### Subfamily Thomisinae Sundevall, 1833

##### Genus Platythomisus

Taxon classificationAnimaliaAraneaeThomisidae

Doleschall, 1859

###### Type species.

*Thomisus 8-maculatus* C.L. Koch, 1845, from Ostindien.

###### Diagnosis.

Sexual dimorphism is distinct in *Platythomisus*. Females can be easily distinguished from most other thomisids by their extraordinary large size [up to 20 mm in length in some specimens of the type species *P.octomaculatus* (C. L. Koch, 1845)] with strikingly contrasting color patterns on the carapace and opisthosoma. Typically, the epigyne has sclerotized margins and a conspicuous epigynal atrium; spermathecae longer than wide, well-sclerotized, and not divided into compartments. The male is much smaller than female (1:3 or more). Palp with VTA, ITA and RTA; tegulum flat, disk-shaped; tegular ridge present; embolus slender.

###### Distribution.

Oriental and African zoogeographic regions.

##### Platythomisus octomaculatus

Taxon classificationAnimaliaAraneaeThomisidae

(C. L. Koch, 1845)

**Figure 1.**
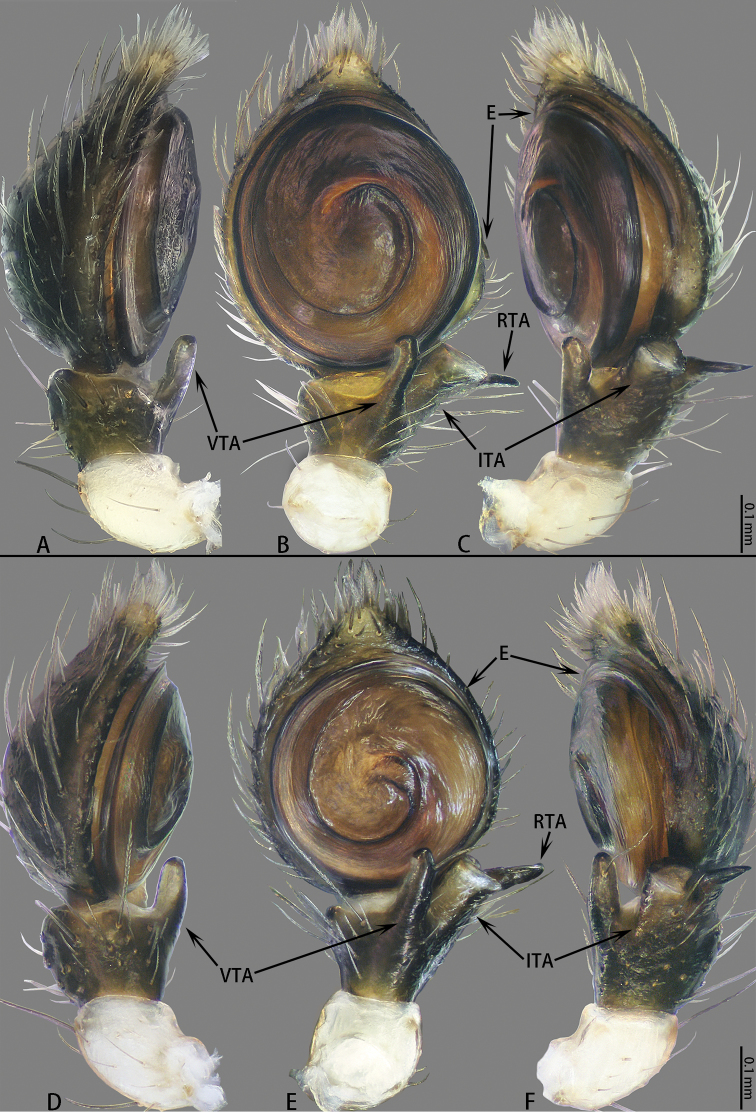
Male palps of *Platythomisusoctomaculatus* (C. L. Koch, 1845) (**A**, **B**, **C**) and *Platythomisusxiandao* sp. nov. (**D, E, F**). **A** Left, prolateral view **B** Same, ventral view **C** Same, retrolateral view **D** Right male palp (Horizontal flip), prolateral view **E** Same, ventral view **F** Same, retrolateral view.

**Figure 2.**
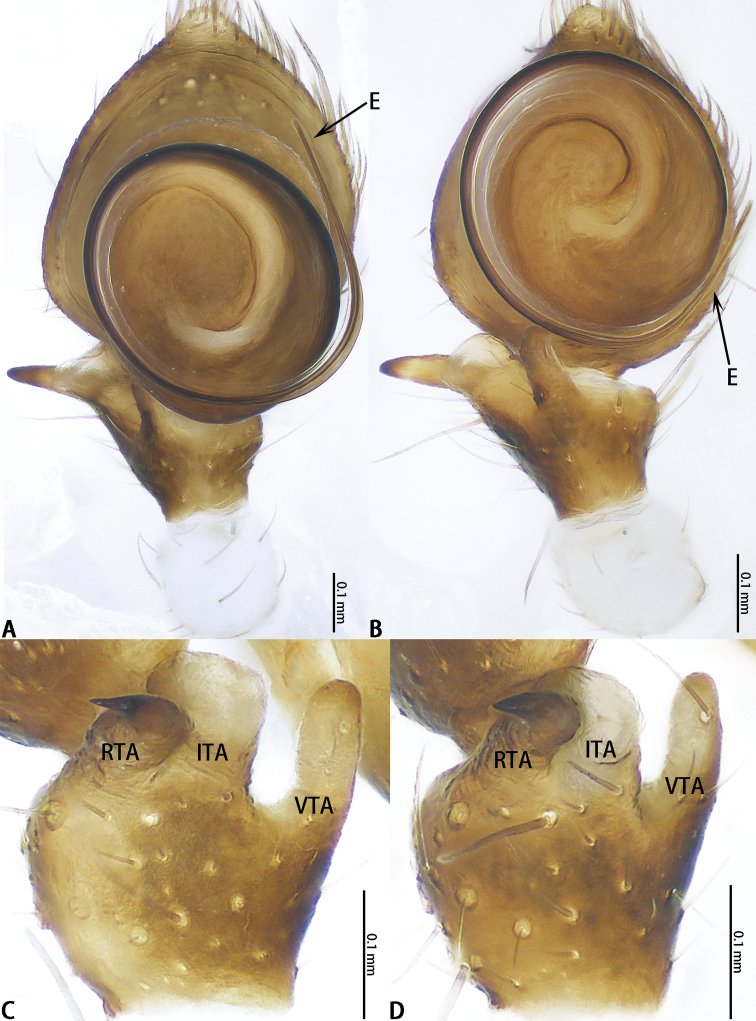
Expanded male palps (treated with 10% KOH) of *Platythomisusoctomaculatus* (C. L. Koch, 1845) (**A**, **C**) and *Platythomisusxiandao* sp. nov. (**B, D**). **A, B** Right, ventral view **C, D** Same, tarsus apophysis retrolateral view.

**Figure 3.**
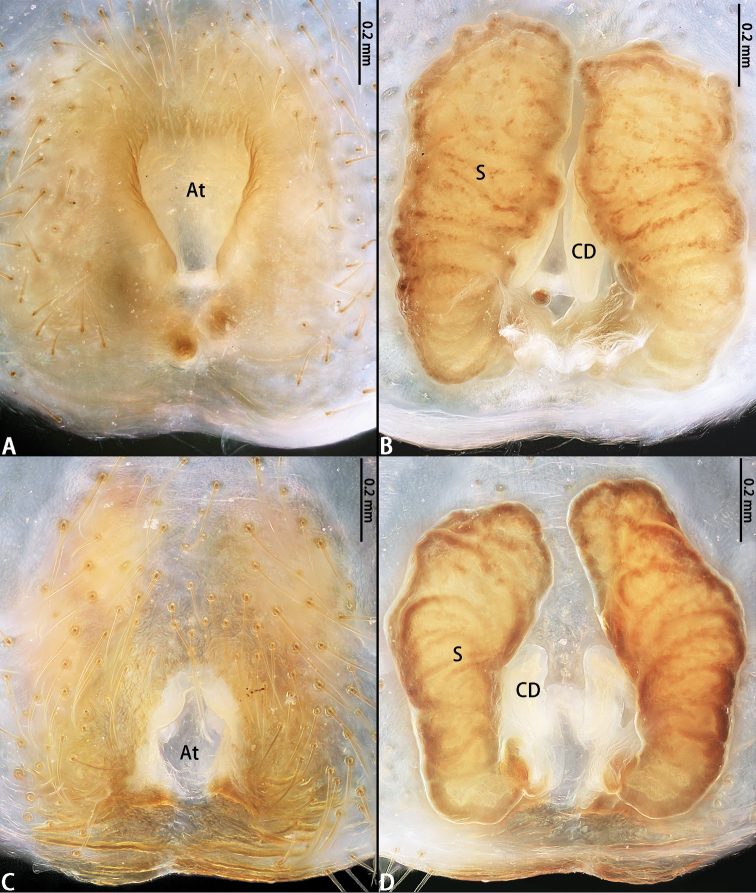
Epigyna and vulvae of *Platythomisusoctomaculatus* (C. L. Koch, 1845) (**A**, **B**) and *Platythomisusxiandao* sp. nov. (**C, D**). **A, C** Epigyne, ventral view **B, D** Vulva, dorsal view.

**Figure 4.**
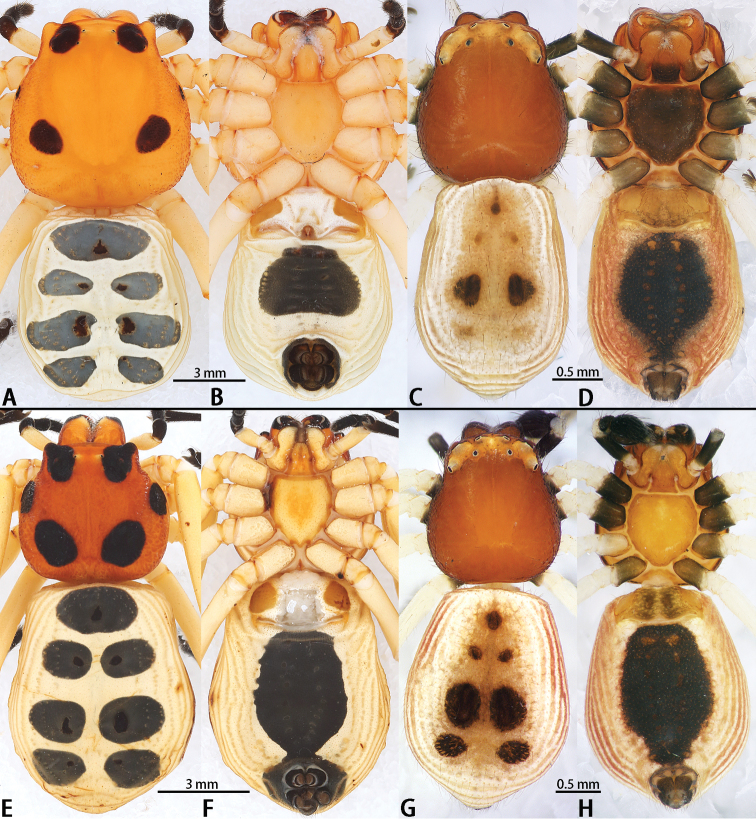
Habitus of *Platythomisusoctomaculatus* (C. L. Koch, 1845) (**A–D**) and *Platythomisusxiandao* sp. nov. (**E–H**). **A, E** Female habitus, dorsal view **B, F** Female habitus, ventral view **C, G** Male habitus, dorsal view; **D, H** Male habitus, ventral view.

**Figure 5.**
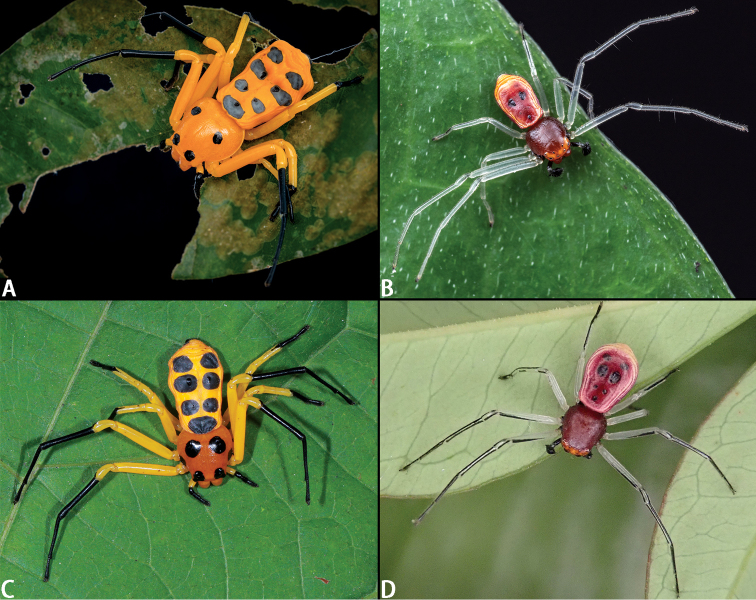
Photos of alive *Platythomisusoctomaculatus* (C. L. Koch, 1845) (**A**, **B**) and *Platythomisusxiandao* sp. nov. (**C, D**) **A, C** Female **B, D** Male.

**Figure 6.**
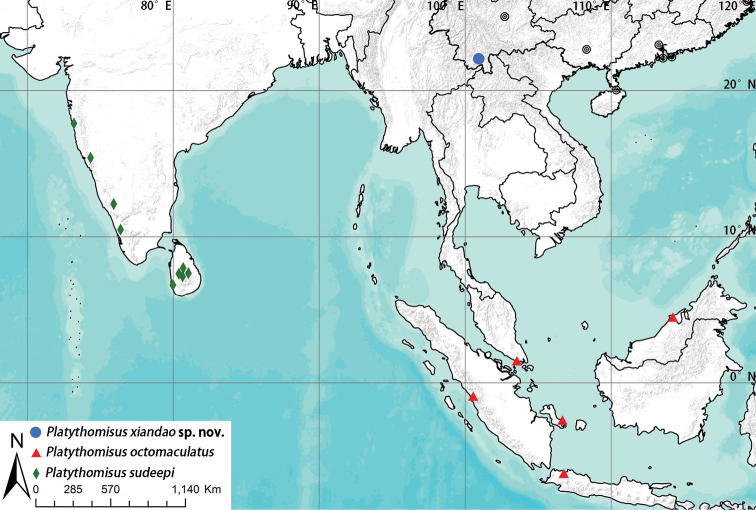
Known distribution of *Platythomisus* species in Oriental Region.

Figs 1A–C , 2A, C , 3A, B , 4A–D , 5A, B , 6

Thomisus 8-maculatus C.L. Koch 1845: 55, fig. 990 (♀).
Platythomisus phryniformis Doleschall 1859: 60 pl. 3, fig. 10 (♀).
Platythomisus octomaculatus van Hasselt 1882: 42, pl. 3, fig. 4 (♀); Simon 1895: 1017, fig. 1076 (♀).

###### Type material.

The holotype of *P.octomaculatus* was not examined as it could not be located in any of the databases of all the major museums in Europe.

###### Specimens examined.

♂ (LKCNHM), Singapore, Pasir Ris Road, Pasir Ris Nature Park, mangrove foliage, 09.X.2018, J. Koh leg.; ♀ (LKCNHM), Singapore, Neo Tiew Crescent, Sungei Buloh Wetland Reserve, mangrove foliage, 01°26'49"N, 103°43'45"E, 20.IV.2016, M. Tan leg. ♀ (LKCNHM), Singapore, Neo Tiew Crescent, Sungei Buloh Wetland Reserve, mangrove foliage, 01°26'53"N, 103°43'42"E, 13.I.2013, J. Koh leg.

###### Diagnosis.

See diagnosis of the species *Platythomisusxiandao* sp. nov.

###### Description.

**Male** (Figs 1A–C, 2A, C, 4C, D, 5B): total length 3.72, carapace 1.62 long, 1.5 wide, opisthosoma 2.18 long, 1.52 wide. Carapace reddish brown. Eye region orange, AER and PER recurved. Eye sizes and interdistances: AME 0.06, ALE 0.09, PME 0.04, PLE 0.08, AME–AME 0.26, AME–ALE 0.20, PME–PME 0.37, PME–PLE 0.28, AME–PME 0.17, ALE–PLE 0.19. Clypeus 0.13 high, red. Chelicerae red, with ridge, without any teeth. Endites and labium red anteriorly and black posteriorly. Sternum black, with sparse hairs. Legs white, coxae faint black, tarsi, metatarsi and tibiae have a longitudinal black line dorsally each. Legs translucent when alive. Leg I: 6.45 (2.05 + 2.26 + 1.23 + 0.91), leg II: 6.60 (2.18 + 2.35 + 1.19 + 0.88), leg III: 3.84 (1.34 + 1.44 + 0.54 + 0.52), leg IV: 3.56 (1.31 + 1.30 + 0.46 + 0.49). Leg formula: 2134. Opisthosoma broadly pentagonal, dorsum yellow-cinnamon, ventrum reddish-brown. Dorsum with three distinct black spots and four smaller faint brown spots, with red folds laterally, ventrum with a large shield-shaped black patch. Spinnerets black.

Male palp (Figs 1A–C, 2A, C). Femur white. Tibia black, VTA club-shaped, with a thick bristle near the apex. ITA boot-shaped, terminally flat with a laterally pointing extension. RTA long, terminal slightly bent. Cymbium black. Tegulum flat, disk-shaped, with a tegular ridge. Embolus spiral, thin, the base of embolus arising from a 1:30-o’clock-position, the length of embolus to the length of embolus base 5:1 (Fig. 2A).

**Female** (Figs 3A, B, 4A, B, 5A) markedly larger and different from male. Total length 17.05, carapace 8.21 long, 7.63 wide, opisthosoma 10.00 long, 7.31 wide. Carapace yellow with three pairs of black spots. The anterior pair cover the ALE and PLE, adjacent to AME and PME; middle spots extremely small. AER and PER slightly recurved. Eye sizes and interdistances: AME 0.14, ALE 0.19, PME 0.10, PLE 0.16, AME–AME 1.14, AME–ALE 0.93, PME–PME 1.61, PME–PLE 1.01, AME–PME 0.54, ALE–PLE 0.42. Clypeus height 0.68, orange. Chelicerae orange, with ridge, margin has a thin brown ring pattern, without any teeth. Endites and labium orange. Sternum orange, with sparsely set hairs. Legs two-colored, with coxa, trochanter, femur and patella in orange, and metatarsus and tarsus black. Coloration of the tibiae varies: black in tibiae I and II, while orange in tibiae III and IV. Leg I: 26.84 (8.14 + 9.62 + 6.02 + 3.06), leg II: 27.24 (8.21 + 9.94 + 6.09 + 3.00), leg III: 15.64 (5.58 + 5.90 + 2.50 + 1.66), leg IV: 15.81 (5.32 + 6.47 + 2.47 + 1.55). Leg formula: 2143. Opisthosoma sub-rectangular with a bluntly pointed posterior end, yellow, laterally pleated. Dorsum with seven blue-grey spots, ventrum with an oblong black patch. Spinnerets black, surrounded by a narrow black ring.

Epigyne (Figs 3A, B) with atrium shaped like a funnel with a broad neck, the length of atrium to the middle width of the atrium 1:1.5, the length of anterior margin to the length of posterior margin 2:1. CD short, approximately half the length of the spermathecae. Spermathecae stout, with almost parallel sides anteriorly.

**Intraspecific variations**: While the holotype has eight spots on the dorsum, as illustrated by Koch (1845), mature specimens seen or photographed in Singapore, Brunei, Thailand, and East and West Malaysia possess only seven dorsal spots but with the eighth spot on the ventrum. In some juvenile females, there are only five dorsal spots on the opisthosoma. The number of spots on the carapace varies between two and four pairs in mature females. The general coloration of live male specimens varies from yellow to red.

###### Distribution.

The type locality “Ostindien”, meaning “East Indies”, refers to Indonesia today. Within Indonesia, the species was recorded in Java as *Platythomisusphryniformis* by Doleschall (1859) and in Padang, Sumatra by Hasselt (1882). However, we failed to find any specimens of the species during field trips in the country. This species has been recorded in Singapore, Brunei, Thailand, and East and West Malaysia (Koh and Leong 2014). A report of *P.octomaculatus* from Assam, India (Yadav et al. 2017) is clearly misidentified. We suggest it may be *P.xiandao* sp. nov.

###### Natural history.

Individuals live among low-lying tree foliage in or around degraded forests and mangrove swamps. Eggs are laid between leaves and sealed with thick silk. The egg sacs are guarded by the mother until the spiderlings hatch in about 2 weeks.

##### Platythomisus xiandao sp. nov.

Taxon classificationAnimaliaAraneaeThomisidae

http://zoobank.org/E45077FE-3209-4887-AD5A-A7DA989FC2FF

Figs 1B–D , 2B, D , 3C, D , 4E–H , 5C, D , 6

###### Holotype.

♂ (IZCAS), China, Yunnan Prov., Jinghong City, Mount Jinuo, hatched from paratype egg sac. Hatched 12.X.2017, matured 10.IV.2018, C.T. Wei leg. **Paratype**: ♀ (IZCAS), same locality data as holotype, collected 06.X.2017, C.T. Wei leg.

###### Etymology.

The specific name is derived from the Chinese word “xiandao” (noun), the name of the Strategic Priority Research Program of the Chinese Academy of Sciences (CAS). The program has made it possible for the biodiversity research team in the CAS to remain as an integral cluster to fulfil all its ambitious goals.

###### Diagnosis.

Male of *P.xiandao* sp. nov. can be easily distinguished from that of *P.octomaculatus* by the length of embolus to the length of embolus base is 3:1 (Fig. 2B), but 5:1 in *P.octomaculatus* (Fig. 2A), the base of embolus arising from a 3:30-o’clock-position in *P.xiandao* sp. nov. (Fig. 1E), and 1:30-o’clock-position in *P.octomaculatus* (Fig. 1B); the atrium of *P.xiandao* sp. nov. is bell-shaped (Fig. 3C) while that of *P.octomaculatus* is funnel-shaped (Fig. 3A); the spermathecae of *P.xiandao* sp. nov. (Fig. 3D) are more slender than those in *P.octomaculatus* (Fig. 3B).

The two species can also be diagnosed by their somatic differences. The males of *P.xiandao* sp. nov. have seven black spots on the opisthosoma (Fig. 4G) and a reddish sternum (Fig. 4H); the males of *P.octomaculatus* have only three distinct black spots on the opisthosoma (Fig. 4C) and a black sternum (Fig. 4D). The females of *P.xiandao* sp. nov. have larger black patches on the carapace (Fig. 4A) and a black patch at the center of ventrum extending posteriorly to connect with the black area surrounding the spinnerets through a narrow neck (Fig. 4F), while the black patch at the ventrum of *P.octomaculatus* females is broadly oblong and well-separated from the spinnerets (Fig. 4B).

###### Description.

**Male** (Figs 1D–F, 2B, D, 4G, H, 5D): total length 3.92, carapace 1.55 long, 1.38 wide, opisthosoma 2.40 long, 1.70 wide. Carapace red. Eye region orange, AER and PER recurved. Eye sizes and interdistances: AME 0.05, ALE 0.09, PME 0.03, PLE 0.06, AME–AME 0.24, AME–ALE 0.17, PME–PME 0.32, PME–PLE 0.28, AME–PME 0.13, ALE–PLE 0.19. Clypeus 0.13 high, red. Chelicerae red, with ridge, without any teeth. Endites and labium red. Sternum red, sparsely set hairs. Legs white, coxa black, tarsus, metatarsus, tibia, patella and femur of leg I and II have two black lines. Legs transparent when alive. Leg I: 5.73 (1.86 + 2.00 + 1.08 + 0.79), leg II: 6.04 (2.02 + 2.10 + 1.10 + 0.82), leg III: 3.20 (1.00 + 1.20 + 0.51 + 0.49), leg IV: 3.27 (1.21 + 1.11 + 0.46 + 0.49). Leg formula: 2143. Opisthosoma oval, white, dorsum cinnamon, ventrum reddish-brown. Opisthosoma dorsum with seven black spots, first one largest, lateral sides with red folds, ventrum reddish-brown, with a large shield-shaped black pattern. Spinnerets black.

Male palp (Figs 1D–F, 2B, D). Femur white. Tibia black, VTA club-shaped, slightly bent, with a bristle near the apex. ITA boot-shaped, terminally flat with lateral extension. RTA long and thin, terminal slightly bent. Cymbium black. Tegulum flat, disk-shaped, with tegular ridge. Embolus slender and spiral, the base of embolus arising from a 3:30-o’clock-position, the length of embolus to the length of embolus base 3:1 (Fig. 2B).

**Female** (Figs 3C, D, 4E, F, 5C) distinctly different from male. Total length 13.01, carapace 5.20 long, 5.05 wide, opisthosoma 8.78 long, 6.35 wide. Carapace yellow with three pairs of big black subcircular patches. The anterior pair extend to the protruding ends on both lateral sides of the ocular tubercles, covering the ALE, PME and PLE, and adjoining the AME. The median pair of black disks smaller than the anterior and posterior pairs. AER and PER slightly recurved. Eye sizes and interdistances: AME 0.11, ALE 0.12, PME 0.05, PLE 0.09, AME–AME 0.81, AME–ALE 0.54, PME–PME 1.02, PME–PLE 0.71, AME–PME 0.37, ALE–PLE 0.36. Clypeus height 0.56, orange. Chelicerae orange, with ridge, margin has a black ring pattern, without any teeth. Endites and labium orange. Sternum orange, sparsely set hairs. Legs bicolored, coxa, trochanter, the femur of leg I and II black, the patella of leg I and II and the femur of leg III and IV orange with a black inverted triangle spot. Other legs orange. Leg I: 18.09 (5.70 + 6.41 + 3.96 + 2.02), leg II: 18.54 (5.96 + 6.54 + 4.00 + 2.04), leg III: 10.82 (3.64 + 4.04 + 1.88 + 1.26), leg IV: 11.16 (3.92 + 4.35 + 1.66 + 1.23). Leg formula: 2143. Opisthosoma pentagonal, yellow. Opisthosoma dorsum with seven black spots, with the largest in the anteriormost spot, lateral folded, ventrum yellow, with a shield-shaped black pattern coalescing with the black ring surrounding the black spinnerets.

Epigyne (Fig. 3C, D) with bell-shaped atrium, the length of atrium to the middle width of the atrium 1:1.4, the length of anterior margin to the length of posterior margin 1:1. CD short, about one-fourth the length of the spermathecae. Spermathecae comparatively slender, shaped like a sea cucumber or hot-dog sausage.

###### Distribution.

China (Yunnan).

###### Natural history.

Individuals of this species hide under the leaves.

###### Remarks.

Based on the 647 bp-aligned sequences, the COI uncorrected K2P-distance between *P.octomaculatus* and *P.xiandao* sp. nov. is 0.073. The result far exceeded the maximum value of intraspecific genetic distance for Thomisidae.

## Supplementary Material

XML Treatment for Platythomisus

XML Treatment for Platythomisus octomaculatus

XML Treatment for Platythomisus xiandao
